# Comparison between Conventional Mechanical Fixation and Use of Autologous Platelet Rich Plasma (PRP) in Wound Beds Prior to Resurfacing with Split Thickness Skin Graft

**Published:** 2015-01

**Authors:** Veena P Waiker, Shanthakumar Shivalingappa

**Affiliations:** Department of Plastic Surgery, MS Ramaiah Medical College and Hospital, Bangalore, Karnataka, India

**Keywords:** Platelet rich plasma, Hemostasis, Skin graft, Edema

## Abstract

**BACKGROUND:**

Platelet rich plasma is known for its hemostatic, adhesive and healing properties in view of the multiple growth factors released from the platelets to the site of wound. The primary objective of this study was to use autologous platelet rich plasma (PRP) in wound beds for anchorage of skin grafts instead of conventional methods like sutures, staplers or glue.

**METHODS:**

In a single center based randomized controlled prospective study of nine months duration, 200 patients with wounds were divided into two equal groups. Autologous PRP was applied on wound beds in PRP group and conventional methods like staples/sutures used to anchor the skin grafts in a control group.

**RESULTS:**

Instant graft adherence to wound bed was statistically significant in the PRP group. Time of first post-graft inspection was delayed, and hematoma, graft edema, discharge from graft site, frequency of dressings and duration of stay in plastic surgery unit were significantly less in the PRP group.

**CONCLUSION:**

Autologous PRP ensured instant skin graft adherence to wound bed in comparison to conventional methods of anchorage. Hence, we recommend the use of autologous PRP routinely on wounds prior to resurfacing to ensure the benefits of early healing.

## INTRODUCTION

Resurfacing of wound beds with split skin graft is the commonest procedure undertaken in the field of plastic surgery. The success of skin graft depends on local vascularity and wound microbiology on one hand and hemostasis and adhesion of skin graft to wound bed on the other. Hemostasis can be achieved by applying epinephrine soaks on the wound bed prior to application of skin grafts, but has both local and systemic side effects.^1^ Skin graft is conventionally fixed to wound margins with sutures, staplers, yano acrylate glue or fibrin glue and quilted to the wound bed in order to prevent shearing and seroma under the graft. However, these methods added to the operating time and cost.^2^^,^^3^

Normal platelet counts in blood range from approximately 1,50,000 to 4,50,000/cum^3^, whereas platelet rich plasma (PRP) contains platelet concentration above baseline compared to same quantity of whole blood. Growth factors^4^ that are released from platelets in the PRP promote angiogenesis, collagen synthesis and epithelization, reduce dermal scarring and facilitate remodelling.^4^

Autologous PRP helps achieve stable haemostasis as it mimics the last steps of coagulation cascade. It brings about instant adhesion of graft to bed preventing any collection under the graft or undue shear.^5^^,^^6^ Chronic wounds may lack growth factors due to decreased production and release, trapping, excess degradation, or a combination of these mechanisms thus delaying wound healing, which is overcome by PRP.^7^^,^^8^ The purpose of this study was to compare two groups of patients with and without topical application of autologous PRP on wound beds prior to resurfacing with split skin grafts. 

## MATERIALS AND METHODS

We hereby present a single center based randomized controlled prospective therapeutic study of Level 1 evidence in two hundred patients during nine months period. The study was approved by the local ethics committee of our hospital. An informed written consent was obtained from all patients. They were divided into two groups of hundred each. In the PRP group, autologous PRP was topically applied on wound beds for graft anchorage whereas in the control group the graft was applied and fixed using conventional methods. 

Acute and chronic traumatic, infective, post burn wounds and wounds following release of post-burn scar contractures were included in the study. Patients with co-morbidities like diabetes and hypertension and those on aspirin analogues were also included in the study. Patients who were positive for HIV, HbsAg, and HCV and those with coagulation disorders and malignancy were excluded.

Rationality for sample size: The study was carried out for finding the efficacy of adhesive nature of PRP compared to staples for fixing the skin graft that was previously reported to be 79.5% versus 59%.^9^ Based on above findings with a power of 85% and confidence level of 95%, it was estimated that 91 patients were needed to be recruited in each arm of the trial. However, 100 patients were recruited into each of the arm.

For randomization procedure, all the 200 subjects were randomly allocated into two equal arms of study by permuting the total sample size. Preparation of autologous PRP was undertaken under anesthesia while the patients were prepped and draped. With aseptic precautions, the blood was drawn preferably from femoral vein alternatively using two 10 ml syringes with anti-coagulant and were transferred to 10 ml vacutainers containing 1 ml CPD-A (freshly obtained from central blood bank). Six milliliter of blood was transferred into each vacutainer by pouring it gently along its walls and mixing by tilting to avoid damage to cells. Blood was then centrifuged at a speed of 1000 revolutions/minute for five minutes (REMI Centrifuge, REMI ELEKTROTECHNIK LIMITED, Vasai -401208, India).

**Fig. 1:**
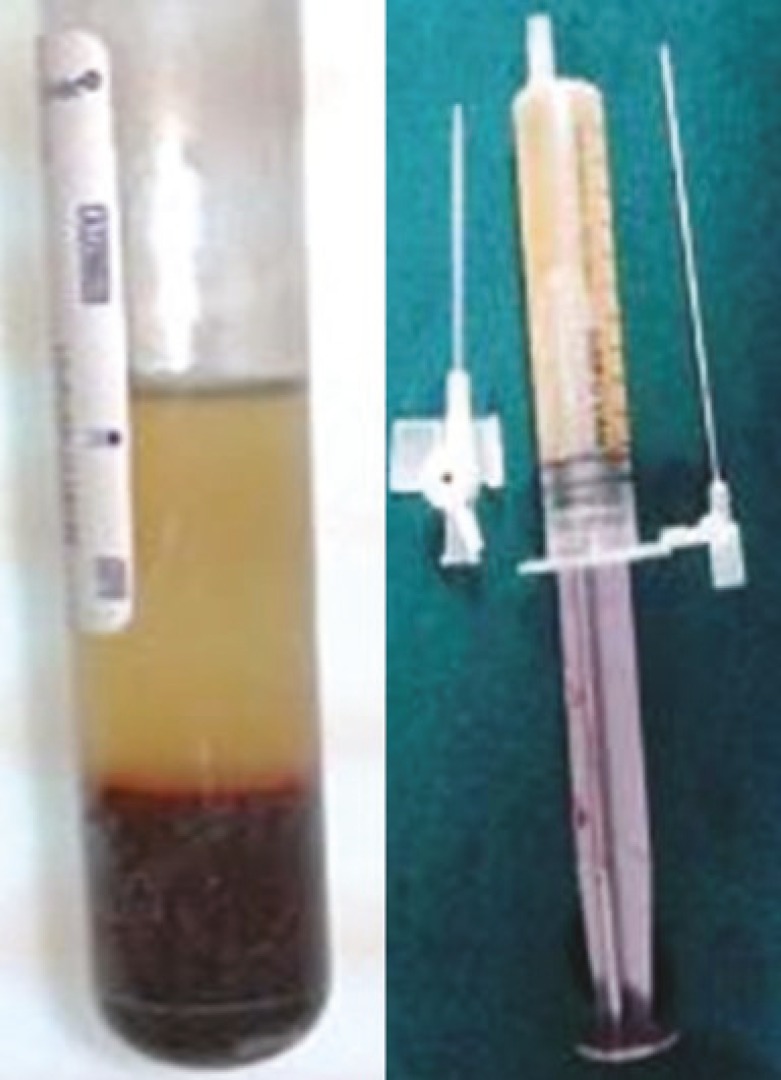
A. Platelet rich supernatant plasma, buffy coat and red blood cells. B. Autologous PRP ready for use.

It was separated into three layers i.e., supernatant PRP, buffy coat known to contain white blood cells and the red cells at the bottom of the vacutainer. Approximately 5 ml of PRP was required for a wound area of 100 sq. cm which was inferred based on the pilot studies. So, the total wound area was calculated and blood collected accordingly. Each vacutainer contained 7 ml (6 ml blood+1 ml CPD-A) that yielded 4-5 ml of supernatant plasma including the buffy coat after centrifugation. The supernatant plasma and the buffy coat was withdrawn into syringes with 16 gauge cannula and kept ready for use (Figures 1A and 1B).

**Fig. 2:**
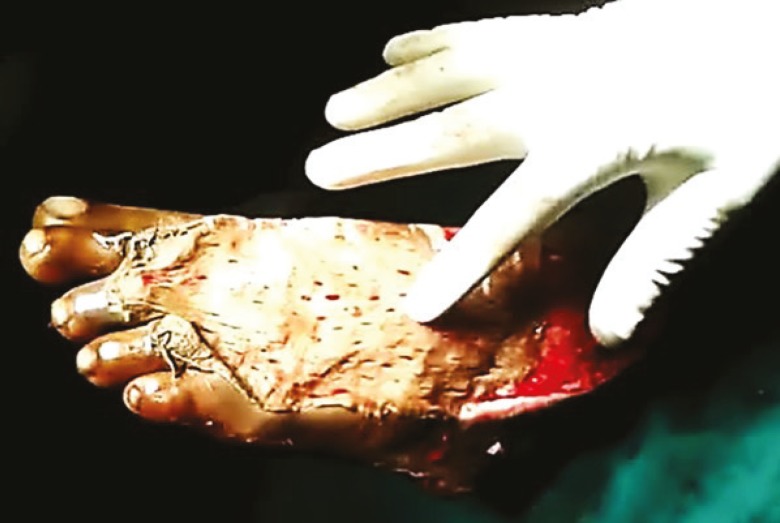
Testing Instant adhesion of skin graft following PRP application by moving finger over the graft on dorsum of left foot.

Aspirin analogues were withdrawn 72 hours before the day of surgery and re-introduced in the post-operative period after 48 hours. Wounds were surgically debrided, hemostasis was secured and lavage was done as the preliminary steps common to both groups. In the PRP group, PRP was topically applied on wound beds through the cannula from the syringe and instant anchorage of skin graft to wound bed was confirmed by moving the graft on the bed with the finger which was done by assistants who were blind to the study (Figure 2). In the control group, sutures or staplers were used to secure the graft to the wound margins and bed. The graft was then covered with a non-adhesive mesh topped with betadine soaked cotton wool and secured with compression or tie over bolus dressings as indicated. 

Conventionally, first graft inspection is done in the early post-operative period i.e., within one week, but the indications of early change of dressing in our study was wetness of outer dressing, odour and pain in both groups. Objective parameters like hematoma and discharge from graft site with significant graft loss, graft edema, frequency of dressings and duration of stay in plastic surgery unit were noted. Moisturisers, massage and pressure garments were advised for a period of eighteen months to prevent lymphedema and scar hypertrophy in both groups. Most of the patients were followed up for a period of three months from the time of discharge to assess scar hypertrophy in the early post-operative period. 

For statistical analysis of data, SPSS software (Version 11.5, Chicago, IL, USA) was used. The proportion of patients revealing instant adhesion, graft edema, discharge from the graft site, hematoma with significant graft loss and scar hypertrophy were calculated for each of the groups. Similar computations were done between control and PRP group with regard to day of first graft inspection, frequency of post-operative dressings and stay in plastic surgery unit. Difference in proportion between two groups was tested through Chi-square test. P≤0.05 was considered for statistical significance.

## RESULTS

Age, gender, etiology of wounds, anatomical distribution, co-morbidities and patients on aspirin analogues were compared in both groups (Table 1). Skin grafts were found to adhere instantly within seconds to the wound bed in all patients of the PRP group which was not seen in the control group. In the control group, 86% underwent first graft inspection and dressing within one week in view of wetness of dressing, pain, hematoma and smell, where some of them required anesthesia for pain control during dressing of large grafted areas. 

**Table 1:** Percentage distribution of age, gender, etiology, regional wound distribution, co-morbidities and medication.

**Parameters**			**Groups**		***P*** ** value**
			**Control (%)**	**PRP (%)**	
Age (years)	0-18		17	13	0.49
	19-36		30	37	
	37-54		23	34	
	55-72		23	14	
	73-90		7	2	
Gender	Male		64	76	0.33
	Female		36	24	
Aetiology	Trauma		24	32	0.27
	Infection		33	41	0.11
	Burns		13	18	0.32
	PBSC		11	8	0.63
	Chronic ulcer		8	12	0.48
Anatomical distribution	Head, neck	10		7	0.61
	Chest	7		9	0.79
	Abdomen	8		5	0.56
	Upper limb	21		19	0.85
	Lower limb	75		83	0.22
Co- morbidities	Diabetes	20		26	0.32
	Hypertension	15		14	0.83
Aspirin analogues	Ecospirin	18		22	0.54

In the PRP group, 95% of patients underwent first post-graft dressing after one week, among them, 94% underwent first graft inspection between 10 and 12 post-graft days and the graft was found to be well adhered and dry. Pain was negligible on the tenth day in the PRP group and hence first graft inspection and dressing of large grafted areas were done without anesthesia. Graft edema was observed in 68% of patients in the control group which was not seen in PRP group. Seropurulent discharge was seen at the graft site in 17% of control patients which was insignificant in the PRP group. There was hematoma under the graft with significant graft loss necessitating secondary grafting in 15% of patients in the control group which was negligible in PRP group. 

Hence, 95% of patients in the PRP group underwent only two post-operative dressings, one on the tenth and the second on the fifteenth day whereas 86% in the control group required frequently (at least 3-5 times) within fifteen days. About 33% of patients were discharged on fifteenth day in the control group, whereas 94% of patients were discharged between 10 and 12 post-graft days in the PRP group (Table 2 and 3). 

**Table 2:** Objective parameters for assessment of efficacy of platelet rich plasma

**Groups**	**Instant adhesion**	**Graft edema**	**Discharge from graft site**	**Hematoma with significant graft loss**	**Scar hypertrophy**
Control (%)	0	68	17	15	25.8
PRP (%)	100	10	2	4	4.7
*P* value	0.001	0.001	0.001	0.008	0.001

The parameters in this table are depicted as percentage distribution in the graph below.

**Table 3:** Objective parameters for assessment of efficacy of Platelet rich plasma

**Groups**	**Control (%)**		**PRP (%) **		**P value**
Day of first graft inspection. (<1 week, >1 week)	86	14	5	95	0.001
Frequency of dressings. (1-2 times, 3-5 times)	14	86	95	5	0.001
Stay in plastic surgery unit. (10 days, >10 days)	33	67	94	6	0.001

The parameters in this table are depicted as percentage distribution in Graphs 1, 2, 3.

In the PRP group, 15% and in the control group, 11% were lost in follow-up after discharge. Four out of 85 patients (4.7%) followed up in the PRP group had scar hypertrophy whereas, 23 out of 89 patients in the control group (25.8%) showed scar hypertrophy. The overall expenditure was 8 times more in the control group when compared to PRP group which was spent on staples/sutures, operating time, change of dressings, secondary grafting and hospital stay. 

**Fig. 3:**
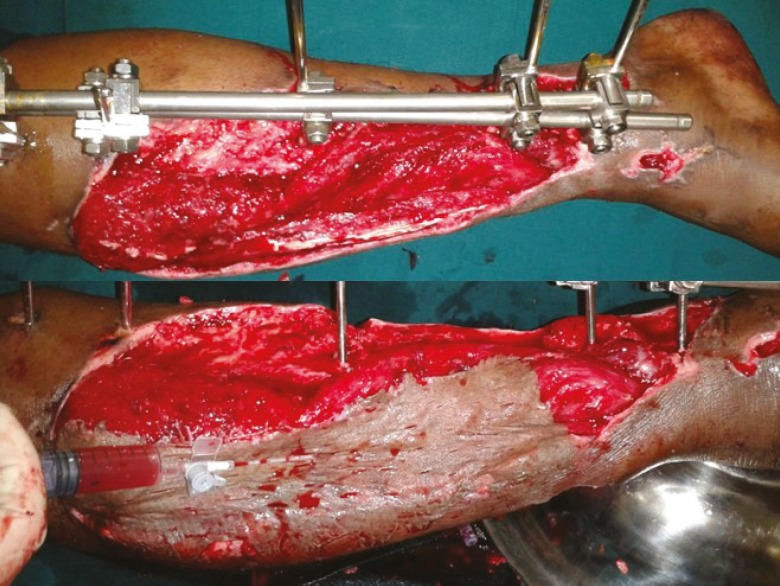
A. Post-excision status of degloved skin in compound injury-right leg. B. PRP application under skin graft.

**Fig. 4:**
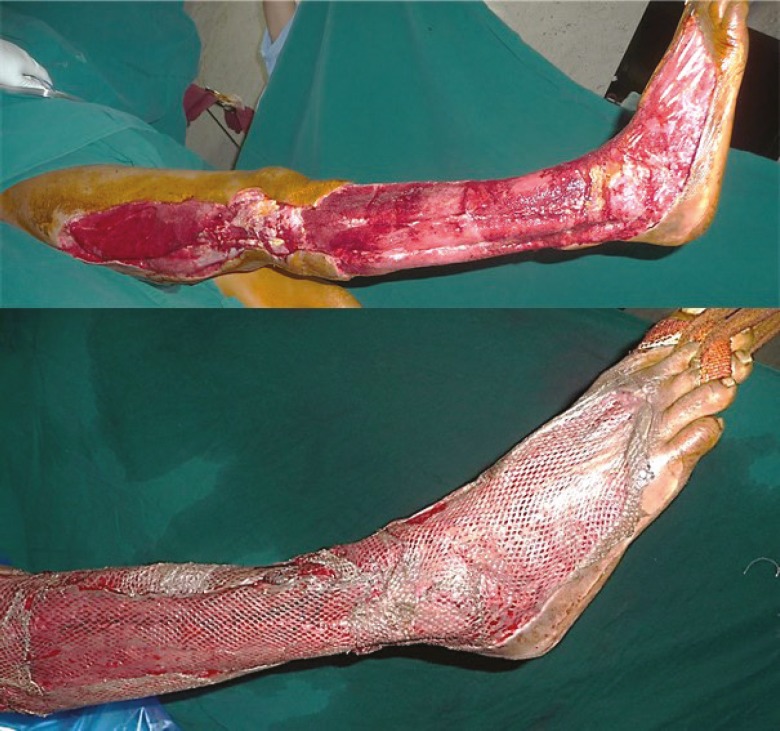
A. Necrotising fasciitis right lower limbpost-debridement status. B. PRP application under the graft

**Fig. 5:**
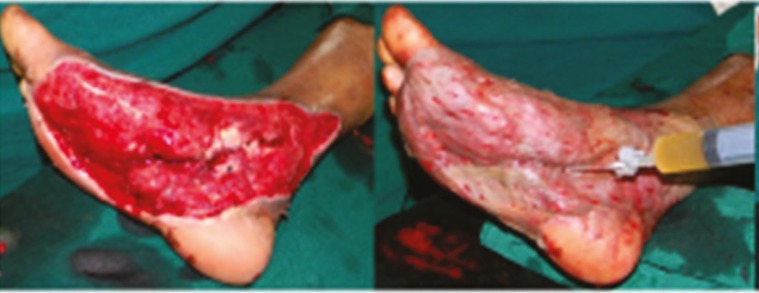
A. Diabetic infection right foot: post-debridement status. B. PRP application.

**Fig. 6:**
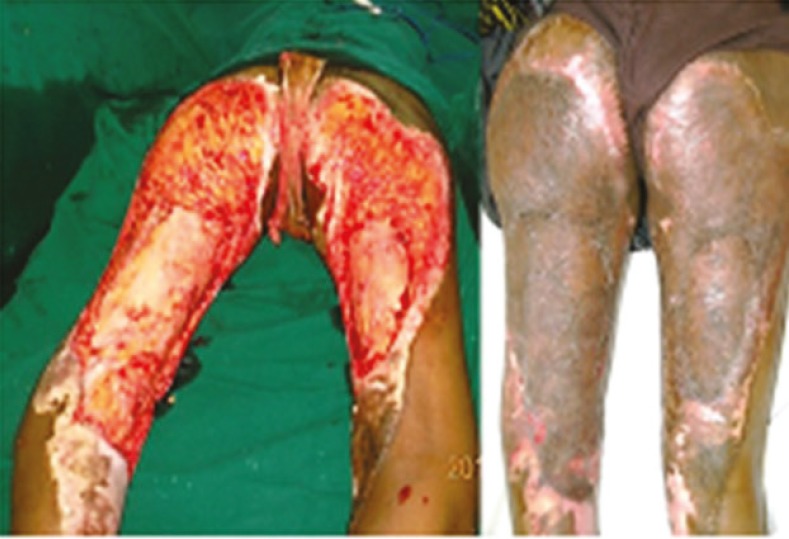
A. 2 year old burn wound on posterior aspect of both thighs and gluteal region following tangential excision. B. 2 weeks post-operative period- well settled skin graft after PRP application

**Fig. 7:**
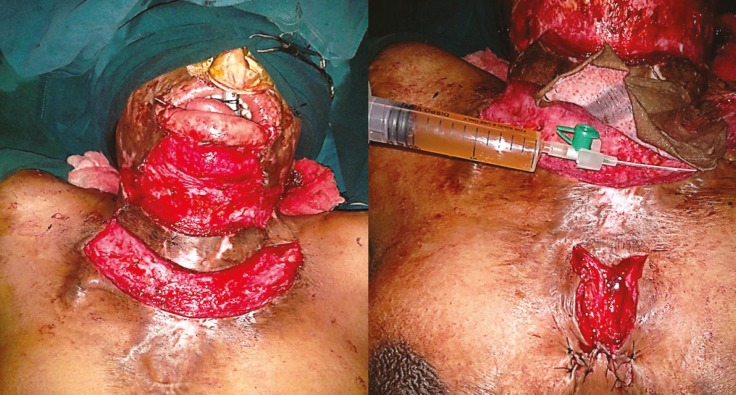
A. Excisional release of post burn labio-mento-sternal scar contracture. B. PRP application under full thickness skin graft

The outcome of all the assessment parameters was found to be statistically significant with a *P* value of 0.05. The used PRP in all types of wounds irrespective of the etiology yielded favorable results (Figures 3A and 3B, Figures 4A and 4B, Figures 5A and 5B, Figures 6A and 6B, Figures 7A and 7B). 

**Fig. 8:**
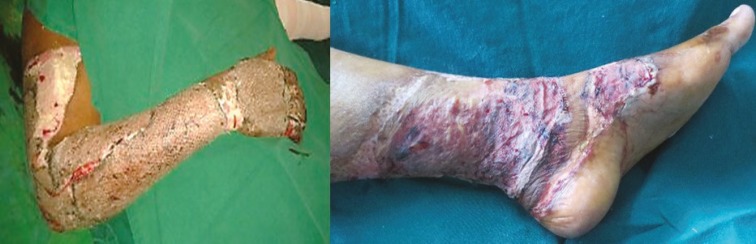
A and B. Showing comparison of non-edematous and edematous skin grafts in PRP and control groups respectively on post-operative Day-3.

**Fig. 9:**
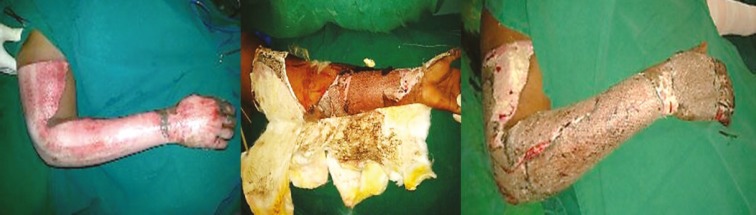
A. Circumferential thermal burns- right upper limb. B and C. Third post-operative day after PRP application showing dryness of inner dressing and firmly adhered non-edematous skin graft

Skin graft edema was noticed up to first two to three days with an increase in weight by 30-50% till the circulation and venous drainage were established in the graft. Good circulation to the graft was found to be restored by 6-7 days post-grafting. In the PRP treated group, we observed only 10% with graft edema, compared to 68% of patients in the control group who had graft edema for more than a week (Figures 8A and 8B). The inner dressings and the skin graft was found to be dry in the PRP group (Figures 9A, 9B and 9C).

**Fig. 10:**
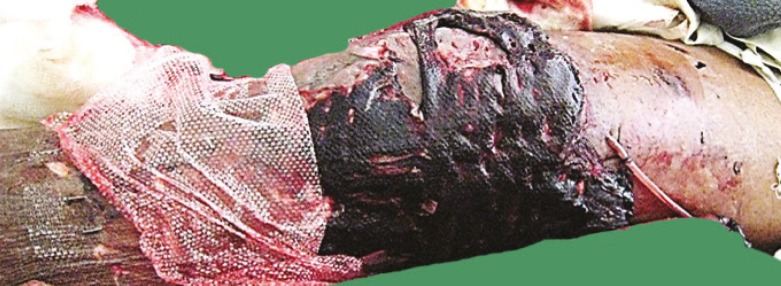
First post-operative day-hematoma under the skin graft left knee and leg in control group

**Fig. 11:**
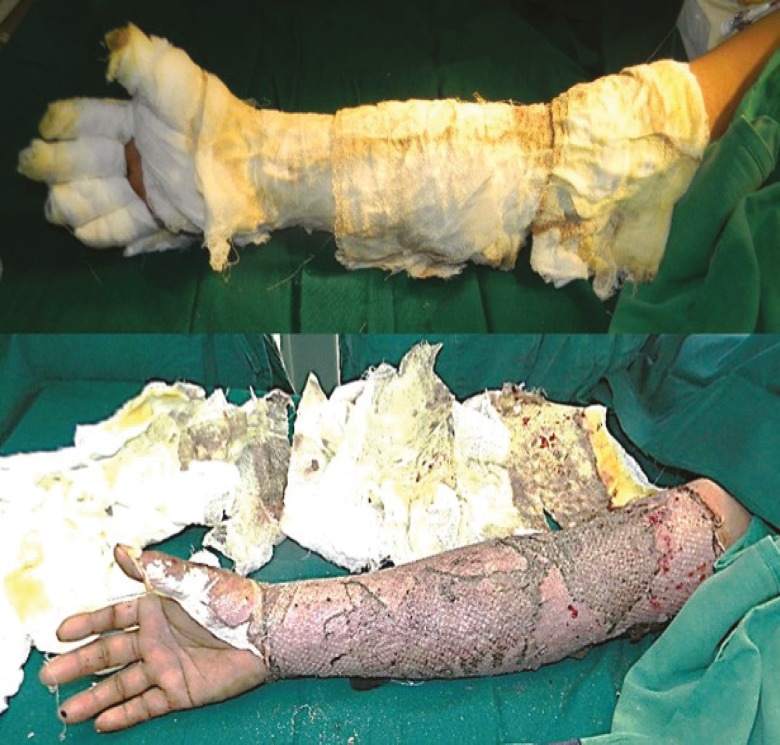
A and B. First graft dressing on post-operative day-10 in post burn wound of right upper limb after use of PRP showing dry and non-edematous graft

Only 4% of the patients in PRP group had hematoma under the graft and required secondary grafting compared to 15% in the control group (Figure 10). Diabetes, hypertension and intake of anti-platelet drugs (oral aspirin) were the relevant factors detrimental to graft take. PRP was beneficial in hypertensive patients and those who were on aspirin analogues in view of its hemostatic properties. In 94% of patients who underwent first graft inspection on the tenth day, grafts were found to be dry, non-edematous and firmly adherent including diabetic patients (Figures 11A and 11B). 

In the control group, 86% underwent frequent dressings within 15 days (3 to 5 times) compared to 95% in the PRP group who underwent only two dressings within 15 days. Reduction in the frequency of dressings is highly appreciated in busy plastic surgery units. The mean hospital stay was 10 days compared to the control group which was 16 days. 

**Fig. 12:**
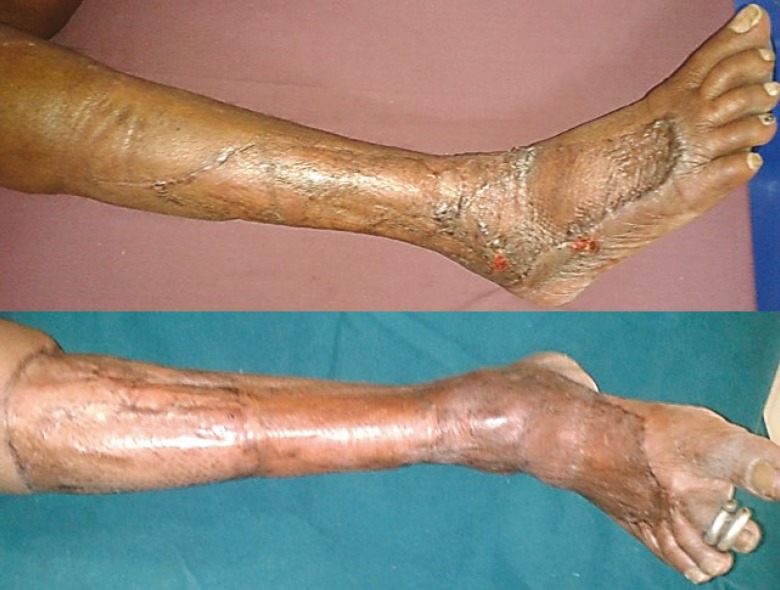
A and B. 3 months post-operative: well settled scar with PRP application

**Fig. 13:**
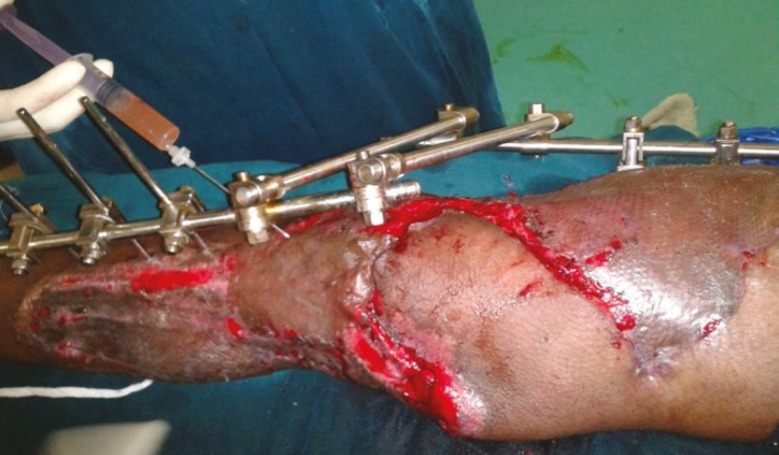
PRP application under the flap in compound injury right leg

**Fig. 14:**
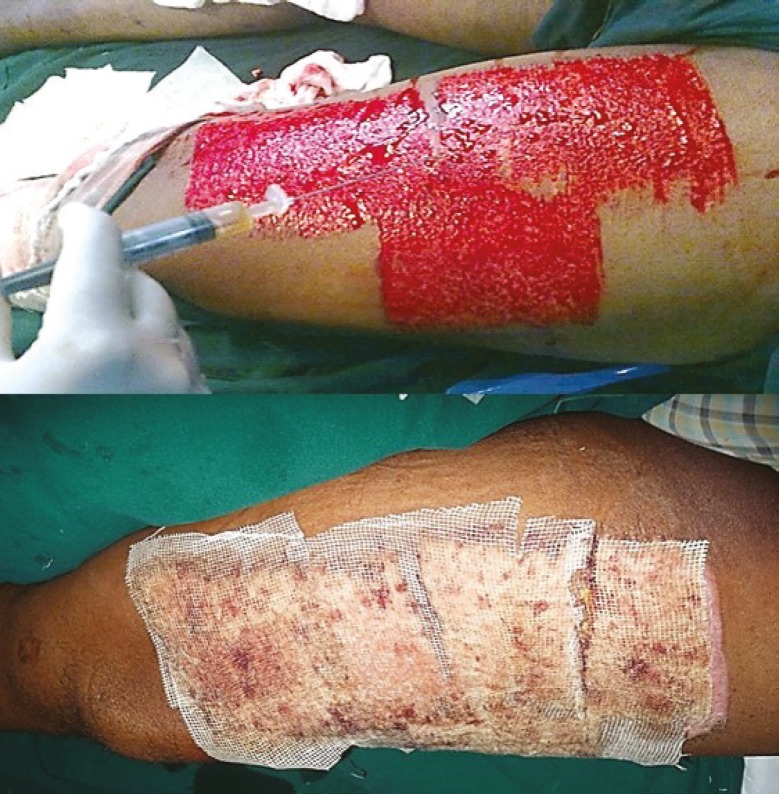
A. PRP on skin graft donor area. B. Post-operative day-3. Wound dry.

In our study higher percentage of scar hypertrophy was found in control group, but in PRP group, scar hypertrophy was not seen probably due to early adhesion, lesser incidence of graft oedema and collection under the graft (Figures 12A and 12B). Our experience on using PRP under full thickness skin grafts, flaps and split skin graft donor areas revealed favourable results (Figure 13, Figures 14A and 14B). The difference in all the objective parameters between controls and PRP groups were statistically significant (*P*<0.05). 

## Discussion

Platelet-rich plasma is defined as a platelet concentration of at least 10,00,000 platelets/µL in 5 ml of plasma. It contains a 3-5 fold increase in the concentration of growth factors.^10^ Proteomic studies have shown that platelets contain over 800 proteins with numerous post-translational modifications, resulting in over 1,500 protein-based bioactive factors.^11^

Our primary aim of application of PRP prior to resurfacing with skin graft is to facilitate its instant stable adhesion to the wound bed without mechanical fixation. We have achieved other benefits like hemostasis, reduction in operating time and frequency of post-operative dressings. Citrate phosphate dextrose-adenine (CPD-A) was the preferred anti-coagulant during preparation of PRP. The citrate binds calcium to create the anticoagulation. The dextrose, buffers and other ingredients are known to support platelet metabolism and viability.^12^

Blood was drawn before the commencement of surgery, as surgery itself lead to platelet activation and the coagulation system.^13^ We used both supernatant plasma and buffy coat in which platelet concentration was more than white blood cells. It is possible that leukocytes could play an important antimicrobial role in PRP, particularly intra-operatively, where the risk of infection is greater.^14^^-^^16^ Platelet activation also results in an increase in anti-inflammatory cytokines due to the presence of hepatocyte GF.^17^

Platelets are activated both by native and exogenous molecules, like collagen, platelet-activating factor, serotonin, calcium, magnesium, thromboxane A2 (TXA2), adenosine diphosphate (ADP), and thrombin.^18^ In PRP preparations, platelets are activated by calcium chloride and/or thrombin for activation, degranulation and release of growth factors. Topical bovine thrombin when used as an activator has been reported to produce fatal coagulopathies due to the formation of antibodies against factor V, factor XI and thrombin.^4^^,^^17^

Calcium chloride is known to induce arrhythmias in some patients.^13^ In view of these side effects reported, we did not activate PRP with either calcium or thrombin but used the fresh preparation on the wound bed as studies have shown that platelet activation also takes place when it comes in contact with collagen in the injured vessel wall.^18^ After preparation with anticoagulant, PRP has been found to be stable for 8 hrs.^6^

Platelet activation results in exocytosis, cytoplasmic α degranulation and an initial burst of growth factors such as insulin-like growth factor 1 (IGF-1), transforming growth factor (TGF), platelet-derived growth factor (PDGF), fibroblast growth factor (FGF), epidermal growth factor (EGF), and vascular endothelial growth factor (VEGF) throughout the platelets life span.^11^^,^^14^^,^^15^

Studies showed the use of PRP in particular etiological groups,^19^^-^^23^ whereas in our study, PRP has been used in all types of wounds irrespective of the etiology and has been found to yield favourable results. The various phases of split skin graft are plasmatic imbibition in first 24-48 hours, second stage of inosculation or capillary ingrowth and lastly the stage of revascularisation. Graft survival in the first two phases is critical to the overall success of graft take.^9^^,^^19^^,^^24^

Applying PRP on wound bed prior to application of skin graft causes hemostasis and provides a sticky surface for instant adherence of graft. Studies showed instant adherence of split skin graft on burn wounds following application of fibrin sealant. There was instant adherence of skin graft to wound bed within seconds in all 100 patients in the PRP test group as compared to control group in whom it did not happen as was seen in other studies but has been compared in a large group of patients in our study.^9^^,^^19^^,^^24^

In view of the adhesive nature of PRP,^25^ the necessity of securing skin graft to wound margins or bed with sutures/bolster sutures, staples or glue was not required in the PRP group as was seen in a study of on forty patients conducted by Gibran *et al.*^9^ This not only saves the operative time but also the surgeon’s time and effort of removing sutures/staplers in the post-operative period. It is known to provide plasmatic nutrition to graft in the early post graft period.^9^

Skin graft edema is generally noticed up to first two to three days with an increase in weight by 30-50% till the circulation and venous drainage is established in the graft. Good circulation to the graft is found to be restored by 6-7 days post-grafting. In the PRP treated group, we observed only 10% with graft edema, compared to 68% of patients in control group who had graft oedema for more than a week. The inner dressings and the skin graft was found to be dry in the PRP group. Platelets are known to stimulate angiogenesis, hence application of PRP accelerates the stage of capillary inosculation and early circulation thereby reducing graft oedema much earlier.^2^^,^^22^

Only 4% of the patients in PRP group had haematoma under the graft and required secondary grafting compared to 15% in the control group. Diabetes, hypertension and intake of anti-platelet drugs (oral aspirin) were the relevant factors detrimental to graft take. PRP was beneficial in hypertensive patients and those who were on aspirin analogues in view of its haemostatic properties. 

In 94% of patients who underwent first graft inspection on the tenth day, grafts were found to be dry, non-oedematous and firmly adherent including diabetic patients. About 86% in the control group underwent frequent dressings within 15 days compared to 95% in the PRP group who underwent only two dressings within fifteen days. Reduction in the frequency of dressings is highly appreciated in busy plastic surgery units. The mean hospital stay was ten days compared to the control group which was 16 days. In our study higher percentage of scar hypertrophy was found in control group. But in PRP group, scar hypertrophy was not seen probably due to early adhesion, lesser incidence of graft edema and collection under the graft similar to other studies.^21^^,^^22^

The authors have the experience of using PRP under full thickness skin grafts, flaps and split skin graft donor areas with favourable results. Larger samples in each etiological group with extrapolation of results and long term follow-ups required to assess the nature of scar are some of the limitations of our study. Future prospects are to precisely assess the circulatory and healing evidences following application of PRP.

In our study we found that the difference in all the objective parameters between controls and PRP groups were statistically significant (*P*<0.05). We found it to be highly beneficial in many aspects both to the patient and surgeon based on our results. Autologous PRP has large potential and practical benefits which improved the outcome of graft take on wounds irrespective of the etiology. 

In view of its safety, low cost, ease of preparation, hemostatic, adhesive and healing properties, it is a boon as adjuvant in the management of wounds mainly in developing countries as it reduced the financial burden. We recommend the use of autologous PRP routinely in all age groups and all types of wounds especially in diabetics and patients on anti-platelet drugs prior to resurfacing to ensure better and faster healing as suggested by our results.

## Note

Please cite this paper as:

Waiker VP, Shivalingappa S. Comparison between Conventional Mechanical Fixation and Use of Autologous Platelet Rich Plasma (PRP) in Wound Beds Prior to Resurfacing with Split Thickness Skin Graft. World J Plast Surg 2015;4(1):50-59.
